# Ursolic acid ameliorates obesity of mice fed with high-fat diet via alteration of gut microbiota and amino acid metabolism

**DOI:** 10.3389/fmicb.2023.1183598

**Published:** 2023-07-06

**Authors:** Chunfeng Tian, Jie Li, Yan Bao, Long Gao, Lixin Song, Kai Li, Ming Sun

**Affiliations:** ^1^School of Public Health, Baotou Medical College, Inner Mongolia University of Science and Technology, Baotou, China; ^2^School of Public Health, Jiamusi University, Jiamusi, China; ^3^Institute of Nutrition and Food Health, Baotou Medical College, Baotou, China; ^4^Baotou Disease Prevention Control Center, Baotou, China

**Keywords:** ursolic acid, obesity, gut microbiota, metabolism, high-fat diet, metabolomics

## Abstract

Obesity has been regarded as one of the major health problems worldwide. Studies demonstrated that ursolic acid (UA) can significantly ameliorate the progress of obesity. However, whether the effect of UA on obesity depends on the regulation of gut microbiota and metabolism is uncertain. To investigate the regulatory role of UA in obese mice from the perspective of intestinal microbiome and metabolomics analyses, an obese mice model was established with a high-fat diet, and the effect of UA on obesity was evaluated. The alterations of gut microbiota and metabolism related to obesity were evaluated by bioinformatic analysis. The results of the gut microbiota analysis showed that UA intervention could shift the Firmicutes to Bacteroidetes ratio at the phylum level and increase in the genera of *Lactobacillus*, *Bacteroides*, and *Akkermansia*. Additionally, metabolomics analysis showed that the beneficial influence of UA on obesity partly depended on amino acid metabolism. The current study demonstrated the roles of UA in the anti-obesity process, which depends in part on alterations in the gut microbiota and metabolism. Therefore, our findings highlight the potential therapeutic effect of UA on the improvement of diet-induced obesity in humans.

## 1. Introduction

Obesity the third most prevalent isepidemiological factor affecting human health after smoking and AIDS. It is also intimately connected to several chronic illnesses, including cancer, type 2 diabetes, renal disease, liver disease, and heart disease, all of which are detrimental to human health (Medanić and Pucarin-Cvetković, 2012; Pan et al., 2018; Ojulari et al., 2019). Therefore, the search for effective solutions to improve obesity is extremely important. There are several preventive or therapeutic strategies for combating obesity, including lifestyle changes, such as diet and exercise, drug treatment, and surgery. However, these approaches can be less satisfactory in the long term, especially drug therapies, which may have serious adverse effects that lead to their successive withdrawal in recent years (Khera et al., 2016; Clément et al., 2018). Therefore, a more effective method should be explored to improve obesity.

The gut microbiota refers to a complex ecosystem in the human digestive tract that includes bacteria, fungi, viruses, archaea, and protists. It could participate in the regulation of multiple physiological functions that maintain the metabolic homeostasis of the host (Qu et al., 2018; Li et al., 2020; Sun et al., 2022). Gut dysbiosis has various adverse effects on the host and may result in metabolic diseases, especially in the obesity condition, which has been revealed by growing evidence over the last two decades. In mice, *Candida parapsilosis* expansion in the gut has been shown to contribute to the promotion of obesity, mainly in terms of higher body weight, accumulation of fungal lipase, and alteration of serum biochemical indicators (Sun S. et al., 2021). Yu et al. (2022) found that the onset of diabetes type 2 mellitus in obese mice was impeded by gut microbiota disturbance, and glucose decomposition may be the primary mechanism by which the jejunal bacteria control the host metabolism. Meanwhile, Wang et al. (2019) demonstrated the numerous metabolic benefits of orally administering live *Parabacteroides distasonis* in high-fat diet (HFD)-induced obese mice, mainly via controlling weight gain, lowering hyperglycemia and hyperlipidemia, and improving hepatic steatosis through the production of succinate and secondary bile acids. According to the findings, effective regulation of host metabolism by intestinal flora is crucial to the development of obesity.

Plant-derived biologically active products have various biological functions in the regulation of a wide spectrum of diseases (Diarra et al., 2016; de Freitas Junior and de Almeida, 2017; Sehrawat et al., 2017). Ursolic acid (UA) is commonly found in fruits and vegetables. According to reports, UA controls a variety of biological functions, including the modulation of several signaling pathways that may stop the onset of chronic diseases (Seo et al., 2018). Recently, the relation of UA to obesity has been documented extensively. Tang et al. (2022) examined the anti-obesity impact of UA in obese rats induced by high fat and streptozotocin via altering insulin and c-Jun N-terminal kinase signaling pathways. Sun A. et al. (2021) demonstrated the crucial role of UA in improving the obesity and metabolic status of obese mice via increasing irisin production, promoting white adipose tissue beigeing, and lowering weight. Moreover, UA has been shown to have potential therapeutic utility in obesity and diabetes via preventing insulin resistance and hyperinsulinemia and by reversing hyperinsulinemia induced by obesity in rats fed with a HFD (González-Garibay et al., 2020). However, the function of UA on the metabolism and gut microbiota during obesity is largely unexplored, so more studies are needed to ascertain whether UA can affect host metabolic pathways.

The objective of this study was to identify the interrelationship among UA, gut microbiota, and metabolites in obese mice induced by diet. 16S rRNA sequencing was conducted to analyze the gut microbiota in mice receiving a HFD, and a metabolomics analysis was used to investigate the mechanism of UA in obesity from the perspective of microbiome changes. This study provides a useful exploration of the potential mechanisms of UA in the regulation of obesity though microbiomics and metabolomics analysis and further demonstrated the beneficial effect of UA on obesity improvement.

## 2. Materials and methods

### 2.1. Animals and diets

In the current research, C57BL/6J male mice ordered form SiPeiFu Biotechnology Co., Ltd (Beijing, China). Then 5-week-old mice were firstly housed under a 12 h light/dark cycle with unrestricted access to food and water, the temperature and humidity of the feeding environment were controlled at 24 ± 1°C and 55 ± 5%, respectively. After 1 week adaptive feeding, the mice were separated equally into three groups for treatment (8 mice per group): (1) control group (fed with the normal diet), (2) HFD group (fed with the HFD), (3) HFD + UA group (fed with the HFD and given a moderate dose of UA, 100 mg/kg/d). The UA was purchased from Xi’an Ruilin Biotechnology Co., Ltd. (Xi’an, China). The normal-fat diet (10 kcal% fat, 20 kcal% protein, and 70 kcal% carbohydrate, catalog no. D12450B) and the HFD (60 kcal% fat, 20 kcal% protein, and 20 kcal% carbohydrate, catalog no. D12492) were Research Diets purchased from Xiaoshuyoutai Biotechnology Co., Ltd. (Beijing, China). During the experiment, the body weight of the mice was record once per week. The treatment were continued for 8 weeks.

### 2.2. Sample collection and preparation

Fresh fecal samples were obtained after an 8-week treatment and stored at −80°C for the gut microbiota and metabolite analyses. After the last treatment administration, mice were allowed with free access to water but no food for 12 h, then were anesthetized via intraperitoneal injection of 1.25% tribromoethanol and the blood samples from the eye orbit were collected. The liver, epididymal white adipose tissue (eWAT), perirenal white adipose tissue (pWAT), and mesenteric fat were stripped and weighed immediately at 4°C after mice were sacrificed, and then maintained at −80°C for the experiment. The collected blood was then centrifuged at 3,000 rpm for 15 min to obtain the serum. After being frozen at −80°C, the serum biochemical indexes were tested using assay kits (Jiancheng, China), including total cholesterol (TC), total triglyceride (TG), high-density lipoprotein cholesterol (HDL-C), and low-density lipoprotein cholesterol (LDL-C). The following formula was used to calculate the index:


Fat/body⁢weight⁢ratio=Visceral⁢fat⁢weight⁢(g)/Body⁢weight⁢(g)⁢×100%

Visceral⁢fat⁢(g)=eWAT⁢weight⁢(g)+pWAT⁢weight⁢(g)+Mesenteric⁢fat⁢weight⁢(g)

### 2.3. Histological analysis of liver

After being treated in 4% formaldehyde solution for paraffin sections, partial liver samples were cut into 5-μm-thick slices for hematoxylin-eosin staining. An optical microscope was used to take pictures of the dyed liver sections (Olympus, Tokyo, Japan).

### 2.4. Bioinformatic analysis of gut microbiota

We extracted genomic DNA from fecal samples using the DNA Extraction Kit (Nobleryder, China) following the manufacturer’s instructions. The 16S rRNA gene’s hypervariable region V3–4 was amplified through PCR using a specific primer. Wekemon Biotechnology Co., Ltd. (Guangzhou, China) carried out the sequencing using the NovaSeq 6000 platform. Quantitative Insights Into Microbial Ecology (QIIME, version 2.0) software was used to obtain quality filter raw sequences. Paired-end reads were assembled using FLASH. Following the detection of chimeras, the remaining high-quality sequences were clustered into operational taxonomic units (OTUs) at 97% sequence identity by UCLUST. A representative sequence was selected from each OTU using default parameters. OTU taxonomic classification was then conducted by BLAST searching the representative sequences set against the Green Genes Database. Based on the emergence of OTUs between groups a Venn diagram was generated using the R package to visualize the shared and unique OTUs between groups. The core-diversity plugin within the open-source QIIME 2 platform was used to calculate the alpha and beta diversity. The specific gut microbiota was analyzed through linear discriminant analysis (LDA) and effect size (LEfSe) analyses. A non-parametric factorial Kruskal–Wallis sum-rank test was used for LEfSe to determine the differences in abundance between groups and used LDA to assess the effect size of each feature, and the threshold on the logarithmic score of LDA analysis was set to 2.0. Spearman’s analysis was used to determine the relationship between microbial communities and serum biochemical parameters based on the relative abundance of microbial species at various taxon levels. All settings were set to default, unless otherwise stated.

### 2.5. Metabolomic profiling analysis

To obtain the supernatant for LC-MS analysis, fecal samples (100 mg) were mixed with extract solution (methanol:water = 4:5, v:v) and centrifuged. A Vanquish UHPLC System from Wekemon Biotechnology Co., Ltd. (Guangzhou, China) was used to perform the LC separation. Orthogonal partial least-squares discriminant analysis (OPLS-DA) was used to compare metabolites in different groups. The variable influence on projection value together with the *p*-value obtained using the two-tailed *t*-test (VIP value > 1.0 and *p*-value < 0.05) were used to select the potential biomarkers. The Human Metabolome Database^1^ was selected to compare the typical MS/MS fragments. The MetaboAnalyst website^2^ based on the Kyoto Encyclopedia of Genes and Genomes (KEGG) database^3^ was used for pathway enrichment analyses.

### 2.6. Statistical analysis

All data were analyzed using one-way ANOVA in SPSS 24.0 (IBM), and Prism 8.0.2 software (GraphPad Software, La Jolla, CA, USA) was used to create all graphics. Spearman correlation was used to examine the correlation between significant microbial communities and metabolism; *p*-value < 0.05 indicated significance.

## 3. Results

### 3.1. Effects of UA administration on physiological, serological, and liver histopathological changes

**FIGURE 1:**
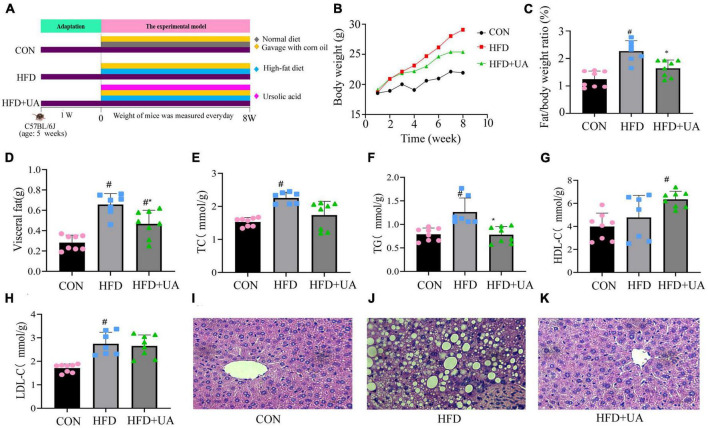
Effect of ursolic acid administration on physiological, serological, and liver histopathological changes. **(A)** Experimental design **(B)** Body weight growth; **(C)** ratio of fat to body weight; **(D)** visceral fat; **(E–H)** Changes in serum biochemical indexes in each group of mice; **(I–K)** hemotoxin-eosin staining of liver tissue sections of mice in different groups (^#^*P* < 0.05 compared to control group, *p < 0.05 relative to the high-fat diet group).

In this study, we investigated the effects of UA on obese mice by constructing an obesity model as shown in Figure 1A. According to Figure 1B, the body weight of mice in the HFD group increased rapidly relative to that of those in the control group over the course of 8 weeks. Nevertheless, UA supplementation decreased this increment (*p* < 0.05). In addition, the HFD could significantly increase the ratio of fat to body weight and visceral fat in contrast to the control, and the UA treatment also reduced the elevated levels but still remained above the normal level (*p* < 0.05) (Figures 1C, D).

Additionally, the levels of TC, TG, HDL-C, and LDL-C were measured to ascertain the potential contribution of UA to the reduction of lipid buildup in mice (Figures 1E–H). The HFD-fed mice had higher TC, TG, and LDL-C levels in serum after 8 weeks of feeding relative to the control group (*p* < 0.05), and these increases were lowered by UA administration, but still remained above normal levels. Interestingly, HDL-C presented high levels of expression in the HFD group relative to those in the control group and remained significantly higher after UA intervention.

Hematoxylin-eosin staining of liver samples was used to assess the triacylglycerol accumulation. The result showed that following the 8-week treatment period, the HFD-fed mice had substantial lipid droplet buildup relative to those in the control group. This accumulation was greatly alleviated after UA intervention (Figures 1I–K), suggesting that UA can successfully limit the buildup of liver lipids and slow the onset of fatty liver in HFD-group mice.

### 3.2. Effects of UA on gut microbiota diversity of mice

**FIGURE 2:**
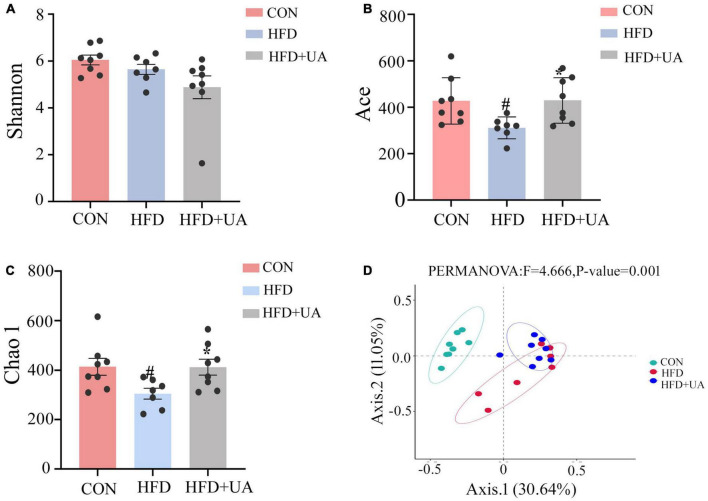
Analysis of alpha and beta diversity of intestinal flora in three groups of mice: **(A)** Shannon index; **(B)** Ace index; **(C)** Chao1 index; **(D)** Principal coordinates analysis (PCoA) (^#^*p* < 0.05 compared to control group, *p < 0.05 relative to the high-fat diet group).

For the 16S rRNA sequencing results included Sparsity curve analysis and OTUs Venn diagram were shown in Supplementary Figures 1, 2. Alpha diversity analysis was used to evaluate the diversity of the microbiota community in each sample, which was presented by diversity indices including Shannon, Ace, and Chao1. The Shannon index reflects the richness and evenness of a community, whereas the Ace and Chao1 indices were positively correlated with community richness (Figures 2A–C). In contrast to those in the control group, the Ace and Chao1 indexes in the HFD group were considerably lower (*p* < 0.05) and greatly increased after UA supplementation. According to the Shannon index, less diversity was present in the HFD group, and there was no improvement as a result of UA supplementation.

Beta diversity analysis in mice was explained using the Principal Coordinates Analysis (PCoA). According to Figure 2D, there was a clear division in the microbiota community among the control and HFD groups, whereas in the HFD and UA treatment groups, the structure of the microbiota community was closer, suggesting that UA and HFD may influence the structure of the gut microbiota.

### 3.3. Effects of UA on the taxonomic composition of the gut microbiota

**FIGURE 3:**
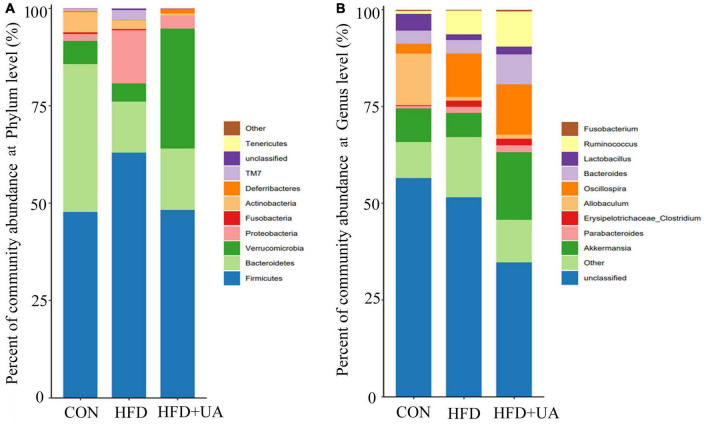
Abundance of intestinal bacteria at the **(A)** phylum and **(B)** the genus levels.

The relative bacterial abundance of the different groups was evaluated to identify specific taxa in the UA treatment group. According to Figure 3A, Firmicutes and Bacteroidetes were the dominant phyla in all groups. HFD treatment significantly changed the gut microbiota composition and structure compared with control group. For example, Firmicutes and Proteobacteria increased significantly, which was accompanied with decreased Bacteroidetes and Verrucomicrobia. Notably, the Firmicutes to Bacteroidetes (F/B) ratio was 0.93 in the control group, and increased rapidly to 2.82 after the HFD treatment, but the changes were reversed after UA supplementation, resulting in a lower F/B ratio of 2.22, which was accompanied by an increase in Verrucomicrobia.

At the genus level, the relative abundance of *Akkermansia*, *Allobaculum*, and *Lactobacillus* decreased and that of *Oscillospira*, *Ruminococcus*, *Parabacteroides*, and *Erysipelotrichaceae-Clostridium* increased in the HFD group (Figure 3B). However, UA supplementation increased the abundance of *Lactobacillus*, *Bacteroides*, and *Akkermansia*, which is inconsistent with the results from the HFD group.

### 3.4. Treatment effects on specific phylotypes in the gut microbiota of mice

**FIGURE 4:**
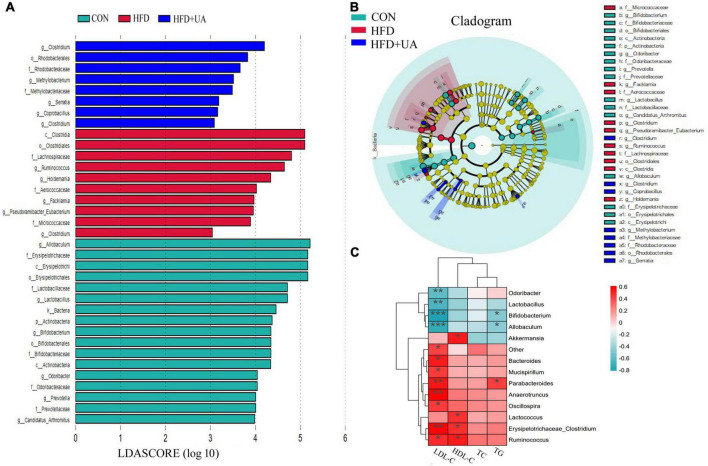
**(A)** Bacterial populations with a linear discriminant analysis score > 2 are displayed. **(B)** Taxonomy cladogram from linear discriminant analysis effect size analysis. **(C)** Heatmap depicting the correlation between major microbial communities and obese-related indicators. *p < 0.05; **p < 0.01; ***p < 0.001.

By using LEfSe to look for variations in the abundance of bacterial taxa between three groups, the features of microbiota in the mice were examined. As presented in Figures 4A, B, 10 significant discriminative features were uncovered in the HFD group and eight were revealed in the UA treatment groups. The indicator microorganisms in the HFD group were identified as belonging to Clostridia, Clostridiales, Lachnospiraceae, Micrococcaceae, Aerococcaceae, *Holdemania*, *Facklamia*, *Ruminococcus*, *Pseudoramibacter-Eubacterium*, and *Clostridium*; likewise, Rhodobacterales, Methylobacteriaceae, Rhodobacteraceae, *Methylobacterium*, *Coprobacillus*, *Clostridium*, *Serratia*, and *Clostridium* were the dominant microbes in the UA treatment group (linear discriminant analysis score > 2, Figure 4A).

### 3.5. Correlations between major microbial communities and biochemical parameters

As shown in Figure 4C, Spearman’s correlation analysis revealed that obesity-related indicators were strongly correlated with the microbial communities. *Parabacteroides*, *Anaerotruncus*, and *Erysipelotrichaceae_Clostridium* were significantly positively associated with LDL-C, and *Bacteroides*, *Mucispirillum*, *Oscillospira*, and *Ruminococcus* were positively correlated with LDL-C. Meanwhile, *Odoribacter*, *Lactobacillus*, *Bifidobacterium*, and *Allobaculum* showed a significant negative correlation with LDL-C. HDL-C was positively correlated with *Akkermansia*, *Lactococcus*, *Erysipelotrichaceae_Clostridium*, and *Ruminococcus*; *Parabacteroides* also showed a positive correlation with TG, but *Bifidobacterium* and *Allobaculum* were negatively correlated with TG.

### 3.6. Effect of UA on metabolic profiles

**FIGURE 5:**
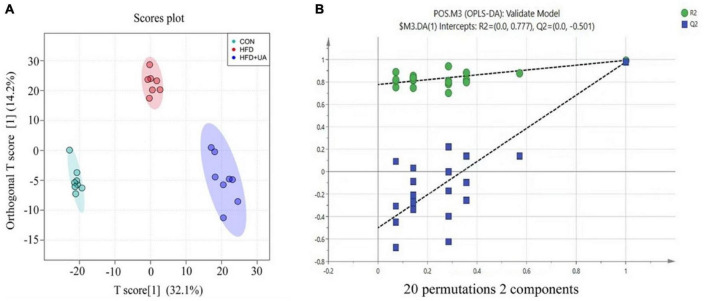
**(A)** Score plots of fecal samples derived from the LC-MS metabolite profiles. Samples from control (green circles), HFD (red circles) and HFD + UA (purple circles) groups. **(B)** Permutation tests were conducted with 20 random permutations in the OPLS-DA model.

To examine the impact of UA on obesity from the viewpoint of host metabolism, the metabolic profiles of mice in the HFD and UA treatment groups were analyzed using non-targeted metabolomics. As shown in Figure 5A, OPLS-DA was conducted to analyze the effect of UA on classification, and the results showed a clear distribution for each group, as the R^2^Y and Q^2^ values of the OPLS-DA score analysis were 0.976 and 0.766, respectively, demonstrating a stable and precise prediction from the existing models. Moreover, the intercepts of R^2^ and Q^2^ were + 0.777 and −0.501, showing that the statistical models are valid and not overfit (Figure 5B).

### 3.7. Changes in metabolites induced by UA

**FIGURE 6:**
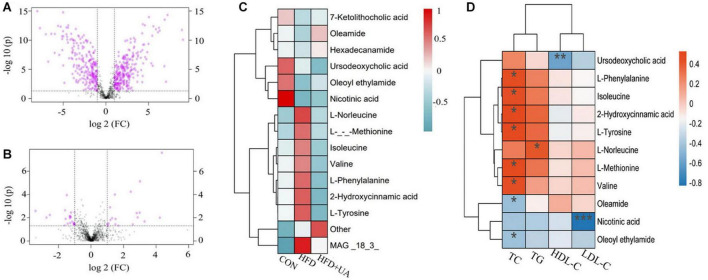
Metabolites in mice feces between groups. **(A)** Univariate statistical analysis of metabolites in fecal samples in the control and HFD groups (multiple > 1 and *p* < 0.05). **(B)** Univariate statistical analysis of metabolites in fecal samples in the HFD and UA treatment groups (multiple > 1 and *p* < 0.05). **(C)** Cluster analysis of the difference in metabolite expression among different groups using hierarchical clustering heatmap. **(D)** Heatmap depicting the correlation between differential metabolites and obese-related indicators. *p < 0.05; **p < 0.01; ***p < 0.001.

Metabolite changes in the feces of mice are presented in Figure 6, showing that the HFD led to 490 metabolite changes (>1-fold, *p* < 0.05) relative to the control group, with 258 increasing and 232 decreasing (Figure 6A). Moreover, the intervention with UA led to 38 metabolite changes (>1-fold, *p* < 0.05) relative to the HFD group, with 18 increasing and 20 decreasing (Figure 6B). These findings indicted that the HFD and UA intervention caused the changes in metabolites in the feces of mice.

A hierarchical clustering heatmap was used to display the relative level of metabolites between three groups. We found that there were eight up-regulated metabolites, mainly including L-norleucine, L-methionine, isoleucine, valine, L-phenylalanine, 2-hydroxycinnamic acid, and L-tyrosine, in mice fed with the HFD relative to those in the control group, and the level decreased after UA intervention. Moreover, there were six down-regulated differential metabolites, 7-ketolithocholic acid, oleamide, hexadecanamide, ursodeoxycholic acid, oleoyl ethylamide, and nicotinic acid in mice fed with the HFD, and the level increased after UA intervention except for that of ursodeoxycholic acid (Figure 6C). Interestingly, correlation analysis of the differential metabolite expression and obese-related indicators using a hierarchical clustering heatmap revealed that the amino acids negatively correlated with UA levels were all positively correlated with TC except for L-norleucine, which was positively correlated with TG. Meanwhile, oleamide and oleoyl ethylamide was negatively correlated with TC, and nicotinic acid showed a significant negative correlation with LDL-C. Notably, these three amino acids also have been showed a positive correlation with UA (Figure 6D). These findings indicated that UA may influence the expression levels of obese-related indicators by regulating amino acid metabolism.

### 3.8. Correlations between gut microbiota and metabolites

**FIGURE 7:**
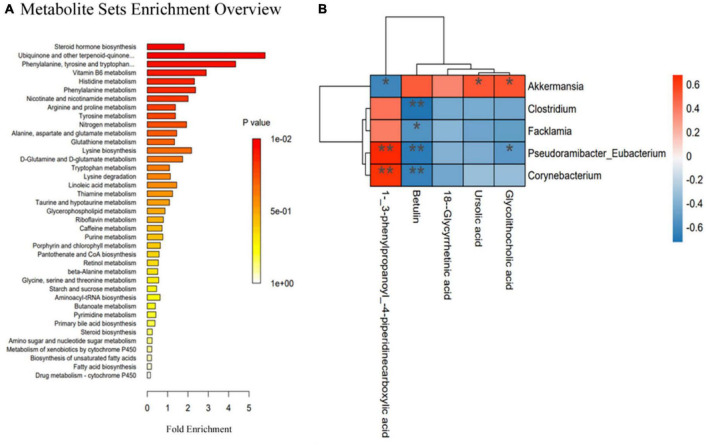
**(A)** Relevant pathways and functions of differential metabolites in Kyoto Encyclopedia of Genes and Genomes analysis; **(B)** Spearman’s correlation heatmap reveals the relationship between the gut microbiota and metabolites. *p < 0.05; **p < 0.01.

To further explore the changes in metabolites after UA intervention, the KEGG database was used for pathway enrichment analysis to identify the relevant pathways and functions of differential metabolites between the HFD and UA treatment groups. According to Figure 7A, the KEGG analysis showed 39 annotated pathways, and the most representative pathways were steroid hormone biosynthesis, phenylalanine, tyrosine and tryptophan biosynthesis, ubiquinone and other terpenoid-quinone biosynthesis, and vitamin B6 metabolism.

Spearman’s correlation analysis was used to reveal the functional correlation of the key microbial phylotypes to the altered fecal metabolites. As shown in Figure 7B, UA and glycolithocholic acid both had a positive correlation with *Akkermansia*, but glycolithocholic acid had a negative correlation with *Pseudoramibacter-Eubacterium*; 1-(3-phenylpropanoy)-4-piperidinecarboxylic acid was positively correlated with *Pseudoramibacter-Eubacterium* and *Corynebacterium* but was negatively associated with *Akkermansia*; betulin had a negative correlation with *Clostridium*, *Facklamia*, *Pseudoramibacter-Eubacterium*, and *Corynebacterium*.

## 4. Discussion

Obesity is regarded as a worldwide public health problem, significantly affecting the health of people in all age groups, and the major factor causing obesity is a high-fat diet (Hariri and Thibault, 2010). UA has multiple biological functions and plays a positive regulatory role in metabolic diseases, especially diabetes and obesity (González-Garibay et al., 2020), but few studies have focused on its impacts on the gut microbiota and metabolites in the disease process. In the present study, we established an obesity model by feeding mice a HFD with or without UA supplement for 8 weeks to explore the effects of UA on the gut microbiota and metabolites in the obesity process.

### 4.1. UA treatment altered obesity-related serum biochemical indicators

We found that several serum biochemical indicators closely related to obesity increased in the HFD group as documented by previous research (Kim et al., 2018; Guo et al., 2020), and the level was rapidly reduced by UA intervention. A previous study demonstrated that UA could decreased the lipid accumulation in the adipose tissues and liver. Meanwhile, the TG and LDL concentrations also decreased while the HDL concentration was increased significantly in mice administrated by UA, which is consistent with our results (Jia et al., 2015). Additionally, The similar results were also reported in other studies that UA treatment significantly decreased the concentration of TG (Li et al., 2014; Chu et al., 2015). Evidence from an animal study indicated that the elevated levels of TC, TG, and LDL-C in serum could improve atherosclerosis and associated cardiovascular disease (Sathyapalan et al., 2018). Moreover, recent research showed that TC, TG, LDL-C and HDL-C could be used as a specific lipid biomarkers to assess the risk of cardiovascular disease among different population groups (Georgoulis et al., 2022). Based on these results, we proposed that UA could regulate lipid metabolism and reduce the risk of atherosclerosis as well as related cardiovascular disease induced by obesity.

### 4.2. General effects of UA on gut microbiota

To explore the effect of UA on the composition of the intestinal flora of obese mice, we analyzed the gut microbiota structure of mice in the HFD and UA treatment groups. It was found that UA intervention shifted the Firmicutes to Bacteroidetes (F/B) ratio in fecal samples of the HFD-induced mice at the phylum level. Recent research demonstrated that Firmicutes has more efficient sugar metabolism relative to Bacteroidetes, which promotes energy absorption and weight gain that contribute to obesity (Bai et al., 2019; Lu et al., 2021; Li F. et al., 2022). Thus, a lower F/B ratio may reduce obesity through energy metabolism, and a similar effect of the F/B ratio on obesity in mice has been further demonstrated in a study that focused on the function of *Rosa roxburghii* Tratt fruit vinegar in the prevention of obesity (Li J. et al., 2022).

The crucial roles of the gut microbiota in obtaining energy from the diet and influencing the pathophysiology of obesity by modulating lipid or glucose metabolism have been demonstrated (Wang and Jia, 2016). In particular, *Akkermansia muciniphila* was shown to participate in the regulation of obesity and could serve as an indicator to evaluate the body’s metabolic status, especially in terms of glucose or lipid metabolism homeostasis (Dao et al., 2016; Wu et al., 2020). Moreover, *A. muciniphila* has a beneficial impact on liver disorders, systemic inflammation, and gut disruption caused by obesity (Xu et al., 2020). Our research found that UA had a positive correlation with *Akkermansia*, and supplementation with UA could significantly increase the abundance of *Akkermansia* relative to that of mice in the HFD group. These findings suggested that the function of UA in the improvement of obesity, at least in part, depends on increasing the abundance of *Akkermansia*, which is beneficial for the steady state of the host’s basal metabolism. In addition, recent research has demonstrated that *Lactobacillus*, as a beneficial bacteria in gut, could alleviate the process of HFD-induced obesity by regulating the intestinal flora (Cai et al., 2020; Hussain et al., 2020; Liu et al., 2022). Our results showed that the abundance of *Lactobacillus* increased after UA intervention, so we further speculated that the anti-obesity action of UA in mice was realized though increasing the commensal gut beneficial microbes and improving the gut barrier integrity.

### 4.3. General effects of UA on amino acid metabolism

Obesity can lead to various degrees of metabolic imbalance, particularly in terms of lipid and carbohydrate metabolism, but amino acid metabolism is also affected. Altered amino acid profiles in the obesity condition are often accompanied by concomitant changes in the resistance and secretion of insulin (Simonson et al., 2020). In particular, the branched-chain amino acids (BCAAs), including leucine, isoleucine, and valine, play vital roles in the pathogenesis of metabolic disorders observed in obesity, and increased BCAA circulating levels are related to insulin resistance (Ridaura et al., 2013; Lynch and Adams, 2014; Pedersen et al., 2016). Mice fed a diet with a lower level of BCAA could quickly lose weight and fat mass before achieving a normal weight, and glucoregulatory control is improved (Cummings et al., 2018). Likewise, ginsenoside Rb1 has been demonstrated to ameliorate glycemic disorder in obese mice partly by decreasing the level of BCAAs, especially isoleucine and leucine (Yang et al., 2021). This provides additional support for our results. Our results showed that UA supplementation could decrease the level of amino acids like BCAAs, tyrosine and others relative to the HFD group, indicating a positive effect of UA on obesity. Furthermore, previous reports showed that BCAAs are partly produced and metabolized by the intestinal microbiome, and obese patients exhibit an imbalanced intestinal microbiome with increased BCAA synthesis and decreased BCAA catabolism (Gill et al., 2006; Liu et al., 2017). Thus, we speculated that the beneficial influence of UA on obesity partly depends on amino acid metabolism and the intestinal microbiome and their interaction.

Notably, besides the BCAAs, obesity risk was also associated with aromatic amino acids, especially tryptophan, which influences host metabolism and has become another potential obesity treatment (Aron-Wisnewsky et al., 2021). As described recently, moderate tryptophan restriction can cause loss of appetite and thermogenesis, whereas severe tryptophan restriction causes hypophagia and weight loss (Zapata et al., 2018). Together with tryptophan itself, metabolites generated from it, including indole, serotonin, and kynurenine, can also contribute to obesity (Konopelski et al., 2019; Pan et al., 2019; Simonson et al., 2020; Aron-Wisnewsky et al., 2021). Additionally, increasing evidence has shown that phenylalanine and tyrosine were also related to the increased incidence of obesity (Libert et al., 2018; Bai et al., 2020; Cheng et al., 2021). Our metabolic analysis showed that the different metabolites could participate in phenylalanine, tyrosine, and tryptophan biosynthesis, further implying that the positive effects of UA on obesity partly depend on its effect on amino acid metabolism.

## 5. Conclusion

In summary, the effects of UA on the microbiome structure and fecal metabolome of obese mice induced by a HFD were investigated. These results indicated that UA intervention not only changed the microbiota composition but also altered host metabolism, in particular that of amino acid metabolism. Our findings could further our understanding of the anti-obesity effects of UA and its potential as a treatment for obesity.

## Data availability statement

The datasets presented in this study can be found in online repositories. The names of the repository/repositories and accession numbers can be found in the NCBI database under accession number: PRJNA960690 (https://www.ncbi.nlm.nih.gov/sra/PRJNA960690), Metabolomics raw data has been uploaded in Metabolights database under accession number: MTBLS7741 (https://www.ebi.ac.uk/metabolights/editor/study/MTBLS7741).

## Ethics statement

The animal study was reviewed and approved by the Ethics Committee of Baotou Medical College.

## Author contributions

CT: investigation, writing–original draft, visualization, and formal analysis. JL: methodology, validation, investigation, and writing–review and editing. LG: conceptualization, resources, supervision, and project administration. YB: investigation, writing–review and editing, and funding acquisition. LS: investigation and supervision. KL: data curation and visualization. MS: supervision. All authors contributed to the article and approved the submitted version.

## Footnotes

1
https://hmdb.ca/

2
https://www.metaboanalyst.ca/

3
https://www.kegg.jp/kegg/pathway.html
