# Supplementary material for: Ursolic acid ameliorates obesity of mice fed with high-fat diet via alteration of gut microbiota and amino acid metabolism
Source: Front Microbiol. 2023 Jul 6;14:1183598. doi: 10.3389/fmicb.2023.1183598 (PMC10359042; doi:10.3389/fmicb.2023.1183598)
Supplement: Supplementary file 1 [file Data_Sheet_1.doc]

Sparsity curve analysis

As shown in Figure S1, with increasing numbers of sequences in the samples, the sparsity curve gradually flattened, meaning that the sequencing depth is reasonable to represent the diversity of the samples. (Figure S1).


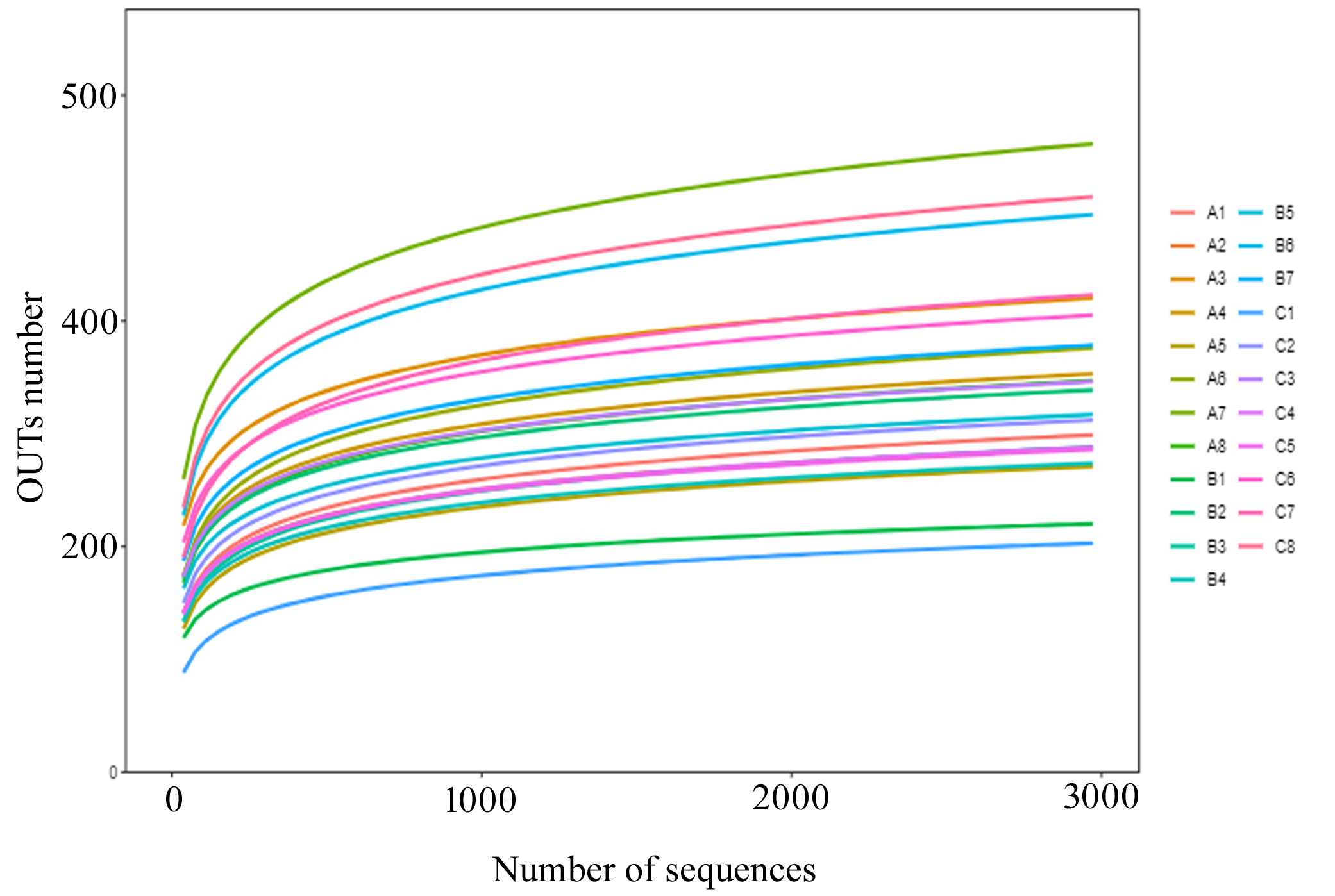


FigureS1 Sparse curves of each sample from three groups of mice

Present the Venn diagram

As shown in Figure S1, a total of 2936 OTUs were detected in the fecal samples of three groups of mice; the number of OTUs in CON, HFD and UA treatment groups was 1432, 1173 and 1450, respectively. Of these, 441 OTUs were shared between the CON and HFD groups, 469 OTUs were shared between the HFD and UA groups, and 603 OTUs were shared between the CON and UA groups. The numbers of OTUs exhibited common between groups and unique to each group.


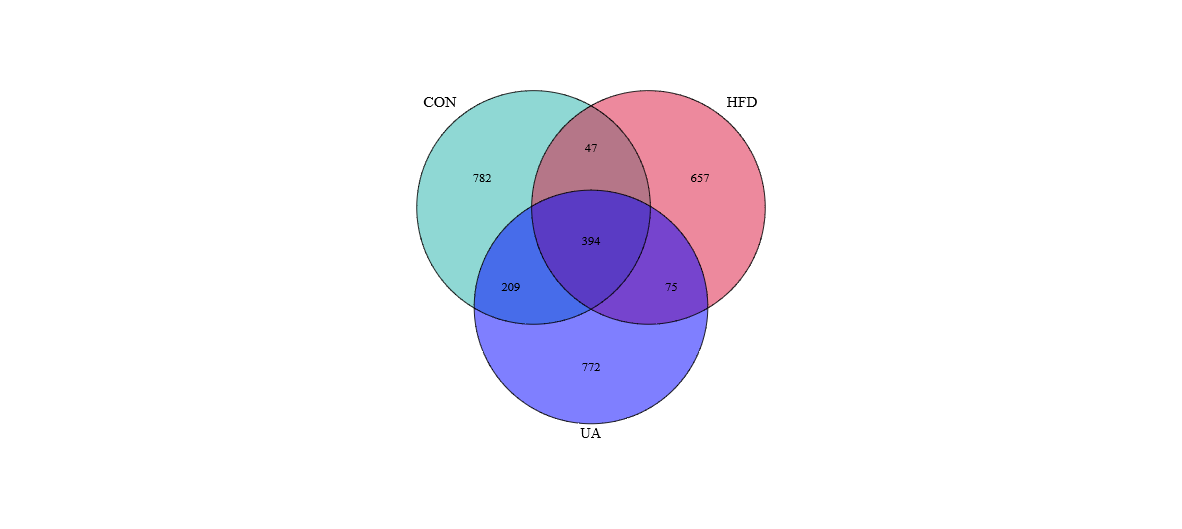


FigureS2 Venn diagram detected in each groups of mice

Raw data

The raw 16sRNA data for this article has been uploaded to the SRA database in the NCBI database and the number is PRJNA960690, (https://www.ncbi.nlm.nih.gov/sra/PRJNA960690), Metabolomics raw data has been uploaded in Metabolights database and the number is MTBLS7741(https://www.ebi.ac.uk/metabolights/editor/study/MTBLS7741).
